# Lovastatin lowers the risk of breast cancer: a population-based study using logistic regression with a random effects model

**DOI:** 10.1186/s40064-016-3606-2

**Published:** 2016-11-08

**Authors:** Rimi Murakami, Chiehfeng Chen, Shu-Yu Lyu, Ching-En Lin, Pei-Chuan Tzeng, Tzu-Feng Wang, Juei-Chin Chang, Ying-Hua Shieh, I.-Fan Chen, Shihping Kevin Huang, Hui-Wen Lin

**Affiliations:** 1 Department of Family Medicine, Wan Fang Hospital, Taipei Medical University, Taipei, Taiwan; 2 Division of Plastic Surgery, Department of Surgery, Wan Fang Hospital, Taipei Medical University, Taipei, Taiwan; 3 Department of Public Health, School of Medicine, College of Medicine, Taipei Medical University, Taipei, Taiwan; 4 Evidence-Based Medicine Center, Wan Fang Hospital, Taipei Medical University, Taipei, Taiwan; 5 Cochrane Taiwan, Taipei Medical University, Taipei, Taiwan; 6 School of Public Health, Taipei Medical University, Taipei, Taiwan; 7 Department of Leisure Industry and Health Promotion, National Taipei University of Nursing and Health Sciences, Taipei, Taiwan; 8 Graduate Institute of Management of Technology, National Chiao Tung University, Hsinchu, Taiwan; 9 Institute of Management of Technology, National Chiao Tung University, Hsinchu, Taiwan; 10 Department of Mathematics, Soochow University, 70 Linhsi Road, Shihlin, Taipei, Taiwan

**Keywords:** Breast neoplasms, Simvastatin, Lovastatin, Fluvastatin, Pravastatin, Atorvastatin

## Abstract

**Background:**

Laboratory studies have demonstrated statin-induced apoptosis of cancer cells, including breast cancer cells, and evidence is accumulating on the mechanism of statin-induced apoptosis. However, despite numerous epidemiological studies, no consensus has been reached regarding the relationship between statin use and breast cancer risk.

**Methods:**

This retrospective case–control study enrolled 4332 breast cancer patients and 21,660 age-matched controls registered in the National Health Insurance program of Taiwan, which covers approximately 99% of the population. The study cases were women for whom a diagnosis of breast cancer (ICD-9-CM code 174.X) had been recorded in LHID2005 between January 1, 2004 and December 31, 2010. A logistic regression model was adjusted for potential confounding factors, including the level of urbanization, and the Charlson Comorbidity Index was applied to assess potential comorbidities. We also considered possible bias caused by random urbanization, because nutrition and lifestyle factors are related to breast cancer incidence.

**Results:**

Our results showed that lovastatin was associated with a lower risk of breast cancer (adjusted OR 0.596; 95% CI 0.497–0.714; *p* < 0.001), and atorvastatin exhibited a protective tendency against breast cancer (adjusted OR 0.887; 95% CI 0.776–1.013; *p* < 0.077).

**Conclusions:**

Although no consensus has been established regarding the relationship between statin use and breast cancer risk, our study indicated that lovastatin is a potential chemopreventive agent against breast cancer. Further detailed research is warranted.

## Background

Breast cancer is the most common cancer affecting women worldwide, in both developed and developing countries. Every year, there are approximately 1.38 million new cases of breast cancer and 458,000 deaths caused by it (International Agency for Research on Cancer 2010). Breast cancer was the most common female cancer in the United Kingdom and United States in 2010 (Jemal et al. 2010), with the lifetime risk of breast cancer for women in the United Kingdom being 1 in 8 (Cancer Research UK 2013). In Taiwan, breast cancer is the fourth leading cancer (in both sexes combined), and the government recommends free mammogram screening for all women over 40 years of age once every 2 years (Health Promotion Administration 2010; Ng 2011).

Studies, including large-scale randomized trials, have investigated potential preventive agents against breast cancer. However, identifying an agent that will be accepted by patients for primary prevention is challenging (Higgins et al. 2012). Statins are widely prescribed for managing chronic hypercholesterolemia and associated morbidities. After patients begin taking statins, they commonly use these agents for a long time (Ahern et al. 2011). If statins were proven to have a protective effect against breast cancer, then this added incentive may increase patient compliance. In high-risk populations, statins may be feasible agents to help prevent breast cancer. Thus, there is increasing interest in the potential effects of statins on breast cancer (Campbell et al. 2006), and proving a connection between statin use and breast cancer risk would have major public health implications.

In this study, the risk of breast cancer is statistically evaluated for statin users and nonusers in Taiwan, as well as for each statin.

## Methods

### Study population and study design

A retrospective case–control design was used. The cases and controls were retrieved from the Longitudinal Health Insurance Database 2005 (LHID2005), which is part of the Taiwanese NHI Database. The NHI program covers approximately 99% of the population of Taiwan, with over 22 million enrollees. LHID2005 comprises a random sample of 1 million of these, and includes data on ambulatory care, inpatient care, demographics, diagnostic codes, prescription details, and the expenses incurred by enrollees.

The study cases were women for whom a diagnosis of breast cancer (ICD-9-CM code 174.X) had been recorded in LHID2005 between January 1, 2004 and December 31, 2010. The ICD-9-CM codes had all been recorded by trained physicians on the basis of pathological evidence. To ensure the validity of the breast cancer diagnoses, only patients who received at least two consistent diagnoses of breast cancer were selected. The study did not include patients only diagnosed once because they may have been too sick or in the late stage of the disease, which was not our study focus. In addition, patients younger than 18 years were excluded from the study. As a result, 4332 breast cancer patients were included in the study.

Controls were selected from among the remaining women in the LHID2005. Women were excluded if they had been diagnosed with breast cancer. The 21,660 participants selected were matched with the cases in a ratio of five to one according to age at the time of diagnosis (18–30, 31–40, 41–50, 51–60, 61–70, and >70 years) and the year at cohort entry from the remaining patients without a diagnosis of breast cancer in the LHID 2005.

### Medication exposure

Exposure to statins and other medications was identified using a drug prescription database. Cases and controls were considered to have been exposed to statins if they received them within the 3 years prior to the index date, which was the date of the breast cancer diagnosis for the cases or, for the controls, the date on which the matched case received a breast cancer diagnosis. The statins included simvastatin (ATC code C10AA01), lovastatin (ATC code C10AA02), pravastatin (ATC code C10AA03), fluvastatin (ATC code C10AA04), and atorvastatin (ATC code C10AA05).

The Charlson Comorbidity Index (CCI) score was assessed according to the 16 Charlson comorbidities (Quan et al. 2005) including myocardial infarction, congestive heart failure, peripheral vascular disease, cerebrovascular disease, dementia, chronic pulmonary disease, connective tissue disease-rheumatic disease, peptic ulcer disease, mild liver disease, diabetes, paraplegia and hemiplegia, renal disease, moderate or severe liver disease, metastatic carcinoma, AIDS/HIV, using diagnoses (excluding cancer) recorded in the NHI before the index date. Other covariates of interest, including age and urbanization level, were obtained for all the cases and controls.

### Statistical analysis

The cluster random effect of urbanization often affects estimates of bias in clinical research. In this study, we took into consideration the bias caused by random urbanization through the following proposed model: ${\text{logit}}({\text{P}}({\text{Y}}_{ij} = 1|u_{i} )) =\upalpha + u_{i} + \beta X_{ij}$, where Y_ij_ = 1 denoted obtained breast cancer, and u_i_ (for *i* = 1, 2, …, 5) denotes a vector of urbanization random effects, which are normally distributed. X denotes covariates of interest in the model. A logistic regression model coupled with a random urbanization effects model, urbanization $u_{i} \sim\,N\left( {0,\sigma_{u}^{2} } \right)$ was used to compare our proposed model with the traditional logistic regression model. The model selection index, Akaike information criterion (AIC), of our proposed model was significantly lower than that of the traditional logistic regression model, indicating the superiority of our proposed model.

Group differences were identified using Pearson’s Chi squared test. Patients were reanalyzed after stratification according to their history of statin use, and a logistic regression with a random effects model was applied after adjusting for potential confounding. All analyses were performed using the R package and SAS statistical package (SAS System for Windows, version 9.1.3, SAS Institute, Cary, NC, USA). A two-tailed *p* < 0.05 indicated statistical significance.

## Results

We assessed data collected from 4332 breast cancer patients and 21,660 age-matched controls. Comorbidities were determined using the CCI (Table 1) and by applying the diagnoses recorded in LHID2005. The patients with breast cancer exhibited higher rates of comorbid dementia (*p* = 0.004), chronic pulmonary disease (*p* = 0.009), mild liver disease (*p* < 0.001), and metastatic carcinoma (*p* < 0.001) than did the controls, as well as a significantly higher CCI score (1.63 ± 1.58 vs. 1.48 ± 1.55; *p* < 0.001). Significantly more patients with breast cancer used statins than did women in the comparison cohort (15.8 vs. 14.6%; *p* < 0.001).

**Table 1:** Baseline variables included demographic characteristics and comorbid medical disorders for two cohorts (n = 25,992)

Baseline variable	Breast cancer patients n = 4332		Control patients *n* = 21,660		*p* value
	No	%	No	%	
*Characteristics*					
Age (years)					
18–30	68	1.6	340	1.6	
31–40	428	9.9	2140	9.9	
41–50	1402	32.4	7010	32.4	
51–60	1306	30.1	6530	30.1	
61–70	721	16.6	3605	16.6	
>70	407	9.4	2035	9.4	
Urbanization level					<0.001
Urban 1	1681	38.8	6857	31.7	
Urban 2	1273	29.4	6348	29.3	
Urban 3	566	13.1	3049	14.1	
Urban 4	455	10.5	2994	13.8	
Urban 5–7	357	8.2	2412	11.1	
*Comorbid medical disorders CCI*					
Myocardial infarction					0.206
Yes	24	0.6	158	0.7	
No	4308	99.4	21,502	99.3	
Congestive heart failure					0.179
Yes	269	6.2	1466	6.8	
No	4063	93.8	20,194	93.2	
Peripheral vascular disease					0.160
Yes	199	4.6	1108	5.1	
No	4133	95.4	20,552	94.9	
Cerebrovascular disease					0.191
Yes	447	10.3	2385	11.0	
No	3885	89.7	19,275	89.0	
Dementia					0.004
Yes	87	2.0	601	2.8	
No	4245	98.0	21,059	97.2	
Chronic pulmonary disease					0.009
Yes	1217	28.1	5665	26.2	
No	3115	71.9	15,995	73.8	
Connective tissue disease-rheumatic disease					0.587
Yes	323	7.5	1564	7.2	
No	4009	92.5	20,096	92.8	
Peptic ulcer disease					0.057
Yes	1470	33.9	7028	32.4	
No	2862	66.1	14,632	67.6	
Mild liver disease					<0.001
Yes	1240	28.6	4849	22.6	
No	3092	71.4	16,762	77.4	
Diabetes					0.065
Yes	945	21.8	4455	20.6	
No	3387	78.2	17,205	79.4	
Paraplegia/hemiplegia					0.420
Yes	99	2.3	543	2.5	
No	4233	97.7	21,117	97.5	
Renal disease					0.188
Yes	180	4.2	1002	4.6	
No	4152	95.8	20,658	95.4	
Moderate or severe liver disease					0.781
Yes	14	0.3	78	0.4	
No	4318	99.7	21,582	99.6	
Metastatic cancer					<0.001
Yes	339	7.8	94	0.4	
No	3993	92.2	21,566	99.6	
AIDS/HIV					1.000
Yes	0	0.0	2	0.0	
No	4332	100.0	21,658	100.0	
CCI score (SD)	1.63	(1.58)	1.48	(1.55)	<0.001
*Medicine use*					
Statins					0.045
Yes	632	14.6	3422	15.8	
No	3700	85.4	18,238	84.2	

Results of the analysis, stratified according to statin use (simvastatin, lovastatin, pravastatin, fluvastatin, or atorvastatin), are given in Table 2. The adjusted odds ratio (OR) for breast cancer among lovastatin users was 0.596 (95% CI 0.497–0.714), which was lower than that for the subjects who did not use lovastatin. Simvastatin, pravastatin, fluvastatin, and atorvastatin did not exhibit a statistically significant protective effect against breast cancer, although atorvastatin showed a protective tendency against breast cancer which did not reach statistical significance (adjusted OR 0.887; 95% CI 0.776–1.013; *p* < 0.077).

**Table 2:** Adjusted odds ratios (OR), and 95% confidence intervals (CIs) for breast cancer in multivariable logistic regression coupled with a random urbanization effects model and stratified by statins

Variable	Presence of breast cancer			
	Case no (%)/control no (%)	Adjusted OR	95% CI	*p* value
*Statins*				
Atorvastatin	319(7.4)/1625(7.5)	0.887	0.776–1.013	0.077
Fluvastatin	143(3.3)/606(2.8)	1.118	0.919–1.356	0.268
Lovastatin	149(3.4)/1122(5.2)	0.596	0.497–0.714	<0.001
Pravastatin	102(2.4)/470(2.2)	1.043	0.831–1.304	0.726
Simvastatin	225(5.2)/1104(5.1)	1.037	0.886–1.215	0.647
CCI scores		1.086	1.061–1.110	<0.001

## Discussion

Our population data revealed a potential protective effect of lovastatin against breast cancer in Taiwanese people. Differences in breast cancer incidence between different ethnicities cannot be ignored. From 2004 to 2008, the incidence rates of female breast cancer in the United States ranged from 84.9 to 125.4 cases per 100,000 women, with the lowest mortality from breast cancer observed in Asian Americans and Pacific Islanders (DeSantis et al. 2011). The association between statin use and breast cancer risk should therefore be evaluated in distinct ethnic populations.

In this study, we found that lovastatin produced a statistically significant protective effect against breast cancer after adjusting for potential confounding factors. Atorvastatin exhibited only a protective tendency against breast cancer. We proposed a logistic regression model coupled with a random urbanization effects model to take into account the potential bias caused by random urbanization, and we used the CCI to evaluate potential comorbidities. We also adjusted for the effect of urbanization, because nutrition and lifestyle are related to breast cancer incidence (Chajes and Romieu 2014).

Our study analyzed the use of five statins based on a large population database of Taiwanese people, and found that lovastatin showed a protective effect against breast cancer. In contrast to the negative findings of other current studies looking at statins and breast cancer risk, the large sample size of our study enhanced the results and made possible the subgroup analysis of each statin. Because of the different chemical properties of lipophilic and hydrophilic statins, most previous studies classified statins into these two groups. However, to prove an association between statin use and the risk of breast cancer requires each statin to be examined individually.

Several studies have suggested reasons to explain why statins are potential chemopreventive agents. One study proposed that statins reduce the production of substances that are crucial for correct localization and translocation to cell membranes; in other words, statins can inhibit molecular signaling pathways (Denoyelle et al. 2001).

In 1990, lovastatin and pravastatin were introduced to the Taiwanese market as the first 3-hydroxy-3-methylglutaryl-coenzyme A (HMG-CoA) reductase inhibitors (statins) (Li et al. 2010). The use of statins in Taiwan rapidly increased with the increasing need to prevent coronary heart disease. Most patients who take statins are likely to be long-term users because of their preventive effects, the main effect being to reduce plasma cholesterol levels by inhibiting a rate-limiting step in the cholesterol synthesis pathway. Statins interrupt the catalysis from HMG-CoA to mevalonate. The reduction of mevalonate contributes to a reduction of geranylgeranyl pyrophosphate (GGPP) and farnesyl pyrophosphate (FPP). These compounds have an important function in molecular signaling pathways in cancer cells. Increasing evidence has revealed that statins have another valuable effect in preventing cancer (Boudreau et al. 2007; Jafari et al. 2013; Ahern et al. 2014). Various laboratory studies have demonstrated the statin-induced apoptosis of cancer cells, including breast cancer cells, with increasing evidence accumulating on the mechanism underlying this (Campbell et al. 2006; Corsini et al. 1995; Dimitroulakos et al. 2001; Niknejad et al. 2007; Woditschka et al. 2010; Ma et al. 2012; Niknejad et al. 2014). Preclinical findings favor the possibility that lovastatin exerts anticancer effects, including effects against breast cancer. However, despite numerous epidemiological studies, no consensus has been established regarding the relationship between statin use and breast cancer risk (Undela et al. 2012). A population-based, case–control study by Boudreau et al. involving 1982 postmenopausal women did not support an association between statin use and breast cancer risk. However, they suggested that long-term statin use (for longer than 5 years) was associated with a slight decrease in the risk of breast cancer (Boudreau et al. 2004). Recently, a population-based case–control study using a nationwide cohort was conducted in Taiwan and found no evidence of an association between statin use and breast cancer (Chan et al. 2014). That study included only patients who were aged at least 50 years, which was a narrower range of age compared to our study. Furthermore, it did not analyze each statin individually. In contrast, Pocobelli et al. reported that patients who used fluvastatin (and no other lipophilic statin) for less than 5 years exhibited a lower risk of breast cancer than did nonusers (Pocobelli et al. 2008). The Women’s Health Initiative cohort study conducted by Cauley et al. reported that users of lipophilic statins (e.g., lovastatin, simvastatin, and atorvastatin) exhibited an 18% lower breast cancer incidence than did nonusers (Cauley et al. 2006). However, after the same cohort was followed up, it was concluded that lipophilic statin users exhibited no significant reductions in breast cancer risk (Desai et al. 2013). Woditschka et al. investigated the association between the risk of specific breast cancer subtypes and lipophilic statin use, and concluded that lipophilic statin users exhibited no reduction in the risk of any breast cancer subtype (Woditschka et al. 2010). Kwan et al. reported that the risk of breast cancer recurrence in all statin users decreased with increasing duration of use after diagnosis (Kwan et al. 2008). In addition, the use of lipophilic statins, particularly simvastatin, was associated with significantly reduced breast cancer recurrence rates in a Danish nationwide cohort study (Ahern et al. 2011). In contrast, one case–control study observed a significantly increased risk of breast cancer in postmenopausal obese women who used lipophilic statins (Eaton et al. 2009). The hypothesis that there is no association between statin use and breast cancer risk has been supported by most observational and epidemiological studies, including three meta-analyses (Undela et al. 2012; Bonovas et al. 2005; Dale et al. 2006). However, organ specificities for the pleomorphic and cholesterol-lowering effects have been observed, although the mechanism for this organ specificity is unknown.

Lovastatin, the first statin isolated (in 1979), has been used clinically for more than two decades and its lipid-lowering and other pleomorphic effects have been thoroughly studied (Corsini et al. 1995; Boudreau et al. 2004). Meanwhile, the effect of lovastatin on apoptosis has been carefully investigated (Corsini et al. 1995). One study examined 59 cell lines for sensitivity to lovastatin-induced apoptosis, and demonstrated that it was tumor specific (Dimitroulakos et al. 2001). The mechanism of lovastatin-induced apoptosis has been reported in several studies. Laboratory studies using mouse models of breast cancer demonstrated that lovastatin inhibits tumor growth and metastasis (Woditschka et al. 2010). Niknejad et al. identified transcription factors that play a crucial role in lovastatin-induced apoptosis in head and neck squamous cell carcinoma cell lines (Niknejad et al. 2007), and multiple stress pathways were found to regulate the cytotoxic effects of lovastatin in squamous cell carcinoma cell lines (Ma et al. 2012). Another study reported that certain substances enhanced lovastatin-induced apoptosis (Niknejad et al. 2014). It has been shown that lovastatin inhibited cell invasion and cell proliferation in breast cancer cell lines (Kang et al. 2009; Klawitter et al. 2010). Thus, preclinical findings support the possibility that lovastatin exerts anticancer effects, including effects against breast cancer cells. However, it should be taken into consideration that cancer cell lines in preclinical studies are exposed to high concentrations of lovastatin, typically ten to one hundred times higher than therapeutic levels (Cho et al. 2011). Organ specificity for lovastatin’s cholesterol-lowering effect has been reported by several studies, and statins have been described as exhibiting organ specificity. Lovastatin inhibits cholesterol synthesis in several organs, whereas pravastatin inhibits cholesterol synthesis mainly in the liver and ileum (Koga et al. 1990; Tsujita et al. 1986). This may explain why each statin exhibits different characteristics in terms of their pleomorphic effects.

## Limitations

Some limitations of our study should be considered. First, although age, urbanization level, and comorbidities using the CCI were adjusted for, we did not examine other confounding factors that may have influenced results of this study, such as age at menarche, age at menopause, a family history of breast cancer, a history of benign breast disease, postmenopausal hormone replacement use, and the mammographic screening status (Eliassen et al. 2005). Second, it is impossible to ensure that all patients who were prescribed statins complied with their treatments. In addition, data about doses and duration were not available. Third, each statin has been available in the Taiwanese market for different periods of time, and these differences may have influenced the statistical results. Fourth, because of the limitation of the time range of the database, which included only the seven years between 2004 and 2010, our study included participants exposed to statins within the 3 years prior to the index date when their breast cancer was diagnosed. However, 3 years may be not long enough to develop or prevent a cancer, and statin users are expected to take the medication lifelong to treat hyperlipidemia. Further study over a longer exposure time may be needed in the future.

## Conclusions

In summary, although no consensus has been established regarding the relationship between statin use and breast cancer risk, our study indicated that lovastatin is a potential chemopreventive agent against breast cancer. Further detailed research is warranted.

## Abbreviations

CCI: Charlson Comorbidity Index
CI: confidence interval
ICD: International Classification of Diseases
LHID2005: Longitudinal Health Insurance Database 2005
NHI: National Health Insurance
OR: odds ratio
